# Phospholipase C-related catalytically inactive protein regulates cytokinesis by protecting phosphatidylinositol 4,5-bisphosphate from metabolism in the cleavage furrow

**DOI:** 10.1038/s41598-019-49156-3

**Published:** 2019-09-04

**Authors:** Satoshi Asano, Yasuka Ikura, Mitsuki Nishimoto, Yosuke Yamawaki, Kozue Hamao, Keiju Kamijo, Masato Hirata, Takashi Kanematsu

**Affiliations:** 1 Department of Cellular and Molecular Pharmacology, Division of Basic Life Sciences, Institute of Biomedical and Health Sciences, Hiroshima University, 1-2-3, Kasumi, Minami-ku, Hiroshima 734-8553 Japan; 2 Department of Biological Science, Graduate School of Science, Hiroshima University, 1-3-1, Kagamiyama, Higashi-Hiroshima, Hiroshima 739-8526 Japan; 3 Division of Anatomy and Cell Biology, Faculty of Medicine, Tohoku Medical and Pharmaceutical University, 1-15-1, Fukumuro, Miyagino-ku, Sendai, Miyagi 983-8536 Japan; 4 Oral Medicine Research Center, Fukuoka Dental College, 2-15-1, Tamura, Sawara-ku, Fukuoka 814-0193 Japan; 5 Department of Cell Biology and Pharmacology, Faculty of Dental Science, Kyushu University, 3-1-1, Maidashi, Higashi-ku, Fukuoka 812-8582 Japan

**Keywords:** Phospholipids, Cytokinesis

## Abstract

Cytokinesis is initiated by the formation and ingression of the cleavage furrow. Phosphatidylinositol 4,5-bisphosphate [PI(4,5)P_2_] accumulation followed by RhoA translocation to the cleavage furrow are prerequisites for cytokinesis progression. Here, we investigated whether phospholipase C (PLC)-related catalytically inactive protein (PRIP), a metabolic modulator of PI(4,5)P_2_, regulates PI(4,5)P_2_-mediated cytokinesis. We found that PRIP localised to the cleavage furrow during cytokinesis. Moreover, HeLa cells with silenced *PRIP* displayed abnormal cytokinesis. Importantly, PI(4,5)P_2_ accumulation at the cleavage furrow, as well as the localisation of RhoA and phospho-myosin II regulatory light chain to the cleavage furrow, were reduced in *PRIP*-silenced cells. The overexpression of oculocerebrorenal syndrome of Lowe-1 (OCRL1), a phosphatidylinositol-5-phosphatase, in cells decreased PI(4,5)P_2_ levels during early cytokinesis and resulted in cytokinesis abnormalities. However, these abnormal cytokinesis phenotypes were ameliorated by the co-expression of PRIP but not by co-expression of a PI(4,5)P_2_-unbound PRIP mutant. Collectively, our results indicate that PRIP is a component at the cleavage furrow that maintains PI(4,5)P_2_ metabolism and regulates RhoA-dependent progression of cytokinesis. Thus, we propose that PRIP regulates phosphoinositide metabolism correctively and mediates normal cytokinesis progression.

## Introduction

Cytokinesis is the final step of cell division, in which a parent cell divides into two daughter cells by the formation and ingression of a cleavage furrow at the plasma membrane after chromosome segregation. A number of phosphoinositides and phosphoinositide-modifying enzymes are involved in cytokinesis, and phosphoinositide production and hydrolysis, which are required for specific steps of cytokinesis, are regulated spatially and temporally^1^. One major phosphoinositide involved is phosphatidylinositol 4,5-bisphosphate [PI(4,5)P_2_]. During cytokinesis, PI(4,5)P_2_ accumulates and is maintained at adequate levels at the cleavage furrow from the initiation of furrowing until the completion of constriction in mammalian cells^2,3^. PI(4,5)P_2_ levels in the plasma membrane are regulated by a variety of metabolic enzymes such as phospholipase C (PLC), phosphoinositide kinases, and phosphoinositide phosphatases including oculocerebrorenal syndrome of Lowe-1 (OCRL1), an inositol polyphosphate 5-phosphatase^4^.

Disruption of PI(4,5)P_2_ levels at the cleavage furrow causes cytokinesis abnormalities. For example, the overexpression of a kinase-deficient phosphatidylinositol 4-phosphate 5-kinase (PIP5K) mutant and of PI(4,5)P_2_-phosphatase synaptojanin or the microinjection of anti-PI(4,5)P_2_ antibodies impairs cytokinesis in mammalian cells^2,5^. Therefore, properly maintaining PI(4,5)P_2_ levels at the cleavage furrow is crucial for normal cytokinesis progression^6^. However, the mechanism by which PI(4,5)P_2_ metabolism is regulated during furrow ingression is not fully understood.

Cleavage furrow progression requires the activation of the small GTPase RhoA, which requires epithelial cell transforming sequence 2 (ECT2), a Rho GDP/GTP exchange factor (RhoGEF)^7^. An important role of PI(4,5)P_2_ is the recruitment of RhoA to the target membrane, as the depletion of PI(4,5)P_2_ inhibits the recruitment of RhoA to the cleavage furrow in cytokinesis^3,8,9^. RhoA has a positively charged amino acid cluster at the carboxy-terminus, which can directly interact with negatively charged phosphoinositides, including PI(4,5)P_2_, *in vitro*^9,10^. ECT2 also interacts with phosphoinositides, including PI(4,5)P_2_, *in vitro*^11^. Therefore, the direct binding of Rho-GEF and RhoA to PI(4,5)P_2_ in the cleavage furrow could be an important process in cytokinesis progression.

Actin filaments and myosin-II are evolutionarily conserved force-generating components of the contractile ring during cytokinesis. Contractile force is generated by non-muscle myosin II, which is regulated by RhoA/Rho-associated protein kinase (ROCK) signalling. Activated ROCK phosphorylates myosin II regulatory light chain (MRLC) directly and also phosphorylates myosin phosphatase-targeting subunit 1, inactivating it. Consequently, phospho-MRLC (Ser19) is augmented^12^, which enhances the ATPase activity of myosin II and promotes the assembly of monomers of myosin II into bipolar filaments, followed by the generation of a contractile force, which then cleaves the parent cell into two daughter cells^13,14^.

PLC-related catalytically inactive protein (PRIP) is a unique protein with high homology to the PLCδ1 isozyme but lacking PLC activity^15–17^. There are two isoforms of PRIP in mammals, PRIP1 and PRIP2: the former is expressed mainly in the brain and the lung, and the latter is expressed ubiquitously^18,19^. PRIP1 was originally isolated as a cytosolic protein containing a pleckstrin homology (PH) domain, which binds to inositol-1,4,5-triphosphate [Ins(1,4,5)P_3_]^16,20,21^. Subsequently, PRIP1 and PRIP2 were reported to bind to PI(4,5)P_2_ via its PH domain and localise to the plasma membrane^22–24^. Although the sequence of the PRIP PH domain is similar to that of PLCδ1, PRIPs exhibit distinct functional characteristics with respect to Ins(1,4,5)P_3_ and PI(4,5)P_2_ binding^25^. Amino acid R40 in PLCδ PH is a critical amino acid residue for Ins(1,4,5)P_3_ binding, and PRIP1 PH(R134Q) mutant (R134 corresponds to R40 in PLCδ PH) fails to bind with Ins(1,4,5)P_3_ or PI(4,5)P_2_^23,26^.

We recently reported that PRIP negatively regulates the conversion of PI(4,5)P_2_ into phosphatidylinositol 3,4,5-trisphosphate by phosphoinositide 3-kinase (PI3K), a process that suppresses the migration of cancer cells^27^. However, little is known regarding the involvement of PRIP in PI(4,5)P_2_-dependent cytokinetic events. Therefore, we examined the potential role and regulatory mechanism of PRIP in cytokinesis.

## Results

### PRIP accumulates at the cleavage furrow during cytokinesis

**Figure 1:**
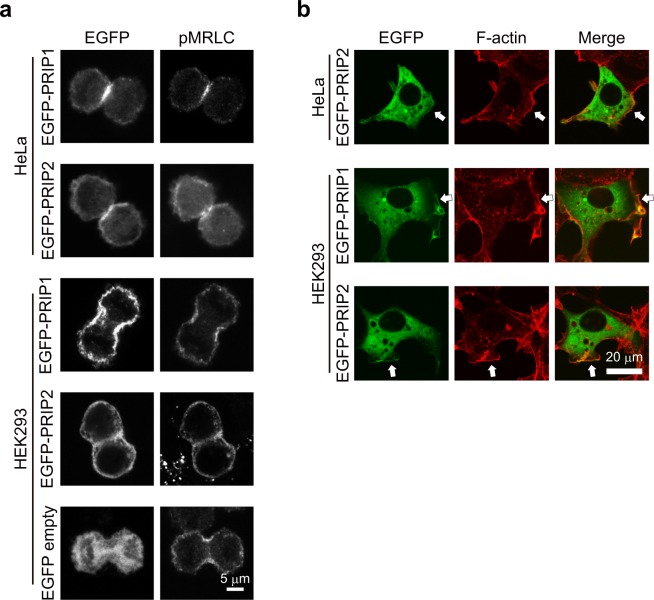
Accumulation of PRIP at the cleavage furrow during cytokinesis. (**a**,**b**) EGFP-PRIP signals were detected in HeLa and HEK293 cells during cytokinesis **(a)** and interphase (**b**). pMRLC **(a)** and F-actin **(b)** were stained with an anti-phospho-MRLC antibody followed by an Alexa Fluor 594-conjugated secondary antibody and ActinRed 555 ReadyProbes reagent, respectively. Images were obtained by confocal microscopy. Arrows in **(b)** show the presence of PRIP on F-actin on the inner face of the plasma membrane. Similar images were satisfactorily obtained from more than three independent experiments.

We first investigated the localisation of PRIP during cytokinesis using HeLa and HEK293 cells transiently transfected with PRIP1 or PRIP2. Both enhanced green fluorescent protein (EGFP)-PRIP1 and EGFP-PRIP2 signals in HeLa cells clearly localised to the cleavage furrow, where phosphorylated MRLC signal (pMRLC; used as a cleavage furrow marker) was observed (Fig. 1a). Similar results were observed in HEK293 cells, in which EGFP signals of PRIP1 and PRIP2 accumulated at the cleavage furrow with pMRLC signal. Some PRIP1 and PRIP2 signals were also observed on the plasma membrane surrounding HEK293 cells, whereas control EGFP-vector signal was observed in the cytoplasm (Fig. 1a).

PRIP1 was originally purified from a cytosolic fraction but has subsequently been purified from a membrane fraction^20,24^. Therefore, we next examined the localisation of PRIP1 and PRIP2 during interphase. EGFP-PRIP1 and EGFP-PIRP2 mainly localised to the cytoplasm, with some signal being observed on the plasma membrane in both HEK293 and HeLa cells (Fig. 1b). These observations suggest that PRIP gravitates toward a component of the cleavage furrow during cytokinesis.

### PRIP participates in the formation and ingression of the cleavage furrow

To examine the role of PRIP at the cleavage furrow, we investigated the cytokinesis machinery regulated by PRIP. We used three cell types in this study: HeLa (a human cervical cancer cell line), HEK293 (a human embryonic kidney cell line), and MCF-7 (a human breast cancer cell line). *PRIP2* mRNA expression was substantial in HeLa and HEK293 cells but minimal in MCF-7 cells according to reverse transcription-PCR analysis. In contrast, *PRIP1* mRNA expression was substantial in HEK293 cells but minimal in HeLa and MCF-7 cells (Supplementary Fig. S1).

**Figure 2:**
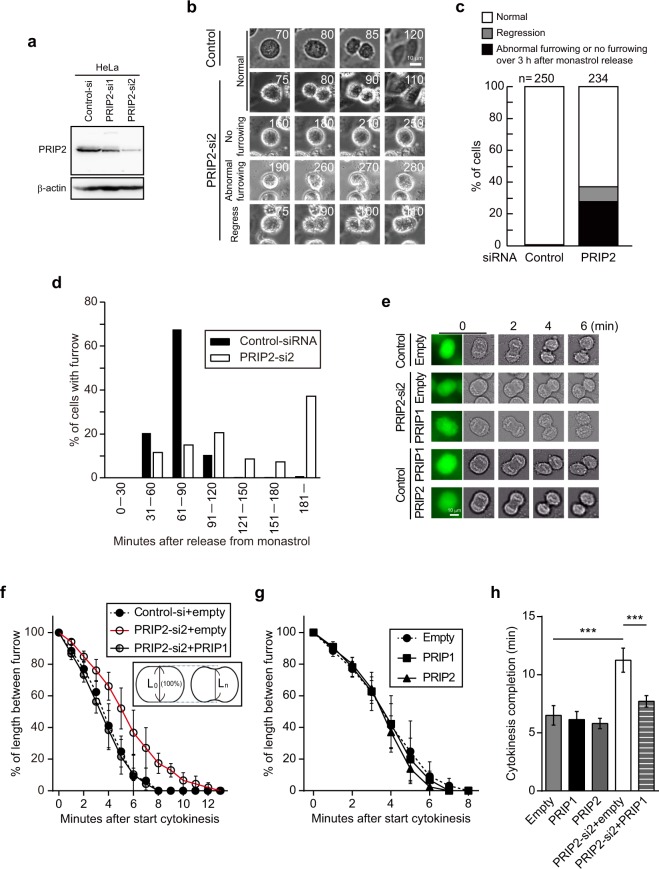
PRIP participates in the formation and ingression of the cleavage furrow. (**a**) Success of *PRIP2* silencing in HeLa cells analysed by western blotting using the indicated antibodies. β-actin was used as a loading control. Control siRNA (Control-si) and *PRIP2* siRNAs (PRIP2-si1 and PRIP2-si2) were used. Each of the original blots is shown in Supplementary Fig. S7a,b. (**b**–**d**) A time course analysis of dividing HeLa cells via time-lapse image analyses. The experiments were repeated at least three times, and a set of representative time-lapse images is shown in **(b)**. The Arabic numerals in **(b)** indicate times (min) after the removal of monastrol from culture media. The frequency of the type of cytokinesis failure (normal: a dividing cell; regression: a cell starting a furrow ingression but not undergoing complete cytokinesis; abnormal furrowing: a cell having asymmetric furrow formation and ingression in the midzone) was analysed in over 230 HeLa cells **(c)**. The distribution of cytokinesis onset time is shown in **(d)**. The y axis of the graph indicates the percentage of cells with furrow measured at each time interval divided by the total mitotic cell number. (**e**–**h**) Aberration of cytokinesis in *PRIP*-silenced HeLa cells and restoration by PRIP gene transfection. Cells were transfected with the indicated siRNAs together with EGFP vector (empty), EGFP-*Prip1* (PRIP1), or EGFP-*PRIP2* (PRIP2). Representative time-lapse series of fluorescence images during furrow ingression are shown **(e)**. The experiments were repeated at least three times. Data are presented as relative furrow ingression [see the schematic diagram in **(f)**]. The y axis of the graphs in **(f)** and **(g)** indicates the percentage of distance (L_n_) between the two constricting poles measured at each time point divided by initial distance (L_0_) at the beginning of cytokinesis. Mean time to the completion of furrowing in **(f**,**g)** is shown in **(h)**. The data are presented as the means ± SD (n > 20 for each group). ***p < 0.001 (Kruskal–Wallis test followed by Dunn’s multiple comparison test).

Endogenous PRIP2 was successfully depleted from HeLa cells, in which *PRIP1* is not expressed, resulting in levels of about 40% and 10% that in control cells following transient transfection with *PRIP2*-specific siRNAs PRIP2-si1 and PRIP2-si2, respectively (Fig. 2a). PRIP2 participation in cell division was analysed using a live-cell imaging technique. HeLa cells transfected with control siRNA displayed normal cell division (Fig. 2b, control), and almost all control HeLa cells underwent cytokinesis within 3 h of synchronisation with and release from monastrol, a potent cell-permeable mitosis inhibitor (Fig. 2c, control). The peak of cytokinesis onset occurred at 61–90 min after release from monastrol (Fig. 2d, control siRNA). By contrast, depletion of *PRIP2* by PRIP2-si2 in HeLa cells retarded the onset of cytokinesis, with no substantial peak of onset apparent during 31–180 min (Fig. 2d). Approximately 60% of PRIP2-si2-transfected cells underwent initiation of cytokinesis within 3 h after release from monastrol (Fig. 2c) and showed normal cell division (Fig. 2b, normal in PRIP2-si2). However, the remaining 40% of cells displayed abnormal cytokinesis and failed to undergo cytokinesis (Fig. 2c). These abnormal phenotypes were classified into no furrowing, abnormal furrowing, and regression (Fig. 2b).

We then analysed the duration of cytokinesis. Dividing HeLa cells exhibited delayed progression of furrow ingression in *PRIP2* siRNA-transfected cells compared with that of controls (Fig. 2e, control/empty vs. PRIP-si2/empty). A plot of cytokinesis speed, which was determined by the rate of change in furrow length, was shifted to the right in cells transfected with PRIP-si2/empty EGFP-vector compared with that in cells transfected with control siRNA/empty EGFP-vector (Fig. 2f). However, this delay was rescued by transfection with EGFP-*Prip1* in *PRIP2*-si2-treated HeLa cells (Fig. 2e,f). The time to completion of cytokinesis was similar among control siRNA-transfected HeLa cells and HeLa cells overexpressing exogenous *Prip1* or *PRIP2* (Fig. 2e, control siRNA-transfected experiments in the upper and lower panels, and Fig. 2g). These data indicate that PRIP overexpression does not affect cytokinesis in HeLa cells; however, *PRIP2* gene depletion inhibits cytokinesis progression, which is ameliorated by the exogenous expression of PRIP (Fig. 2h).

### PRIP regulates MRLC phosphorylation at the cleavage furrow during cytokinesis

**Figure 3:**
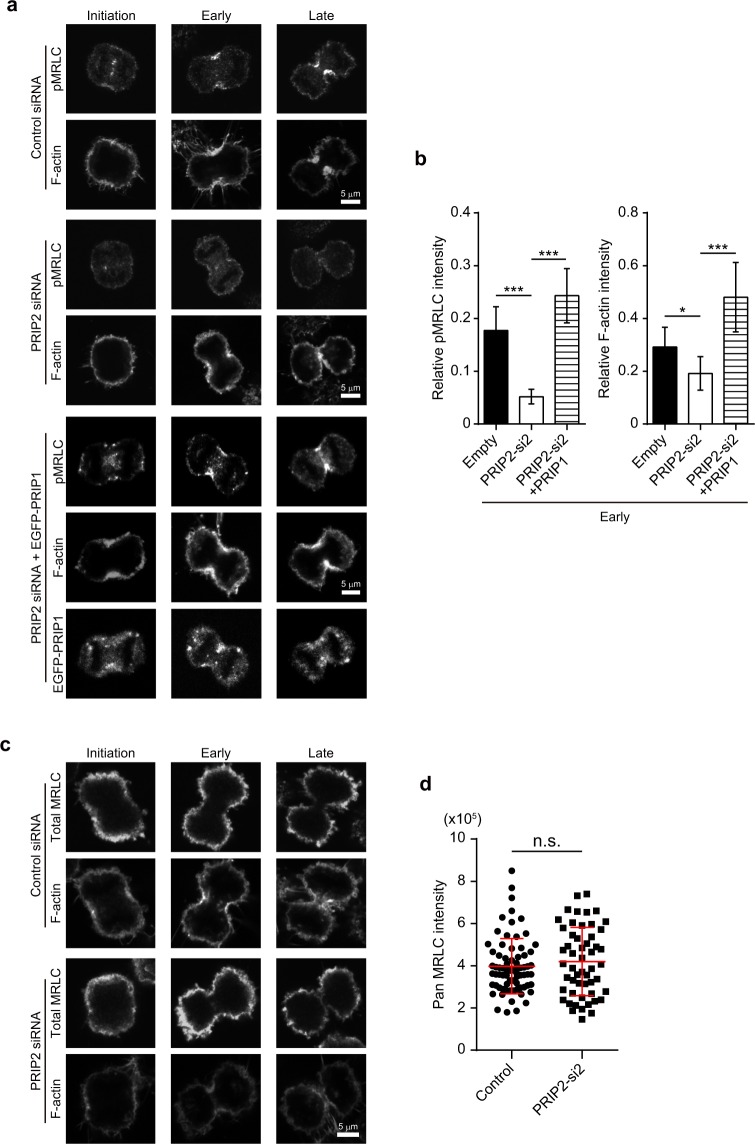
PRIP regulates MRLC phosphorylation at the cleavage furrow during cytokinesis. **(a**–**d)** HeLa cells were transfected with a control siRNA (**a**,**c**, upper panels), PRIP2-si2 (**a**, middle panels; **c**, lower panels), or PRIP2-si2 together with EGFP-tagged *Prip1* (**a**, lower panels). Phosphorylated MRLC (pMRLC) **(a**), pan MRLC **(c**), and F-actin **(a**,**c)** were stained with an anti-phospho-MRLC antibody, anti-total MRLC antibody, and Alexa Fluor 350-labelled phalloidin, respectively. The images were obtained by confocal microscopy. Representative set of images is shown during cytokinesis [initiation phase, and early and late stages in furrow ingression] in **(a**,**c)**. Graphs in **(b)** show the relative fluorescence intensity of pMRLC (left panel) and F-actin (right panel) at the cleavage furrow in an early stage of furrow ingression. The relative fluorescence intensity (y axis) was defined as the fluorescence intensity of pMRLC or F-actin in the cleavage furrow divided by the total intensity of pMRLC or F-actin in the cell, respectively. The data are presented as the mean ± SD (n = 46 for each group). *p < 0.05, ***p < 0.001 (Kruskal–Wallis test followed by Dunn’s multiple comparison test). Graph in **(d)** shows the fluorescence intensity (arbitrary units) of pan MRLC at the cleavage furrow. The data are presented as the means ± SD (n > 60 for each group). n.s.: not significant (Student’s *t*-test).

An actomyosin-based contractile ring is present at the cell equator. Actin filaments and non-muscle myosin II are components of the contractile ring during cytokinesis, and these generate the constricting force. The phosphorylation of MRLC at Ser19 activates the ATPase activity of myosin II, which promotes binding to and motility along actin filaments^28^. To investigate whether PRIP affects the localisation of pMRLC to the cleavage furrow, we performed immunocytochemistry with an anti-pMRLC antibody and stained F-actin with phalloidin. pMRLC signal was detected at the cleavage furrow in control siRNA-transfected HeLa cells during the initiation phase and the early and late stages of furrow ingression (Fig. 3a, upper panels, control siRNA). However, transfection of HeLa cells with *PRIP2* siRNA reduced pMRLC and F-actin signals at the cleavage furrow compared with those in the controls (Fig. 3a, upper vs. middle panels). In experiments involving co-transfection with a full-length *Prip1* gene and *PRIP2* siRNA, EGFP-PRIP1 localised at the cleavage furrow and rescued the localisation of pMRLC to the furrow (Fig. 3a, middle vs. lower panels). Next, the signal intensities of pMRLC and F-actin at the cleavage furrow were assessed by dividing each furrow intensity by the corresponding total cell intensity in the early stage of furrow ingression (Fig. 3b). The fluorescence intensities of the two signals in *PRIP2*-silenced cells were restored by *Prip1* co-transfection.

The pan MRLC signal localised on the plasma membrane in control cells during cytokinesis, and this was not altered in *PRIP2*-silenced HeLa cells (Fig. 3c). Moreover, the pan MRLC signal at the cleavage furrow did not differ between control siRNA and *PRIP2* siRNA groups (Fig. 3d). These data suggest that PRIP participates in the phosphorylation of MRLC at the cleavage furrow without affecting MRLC accumulation at the furrow.

### A phospho-mimic MRLC mutant rescues delayed cytokinesis progression in *PRIP*-silenced HeLa cells

**Figure 4:**
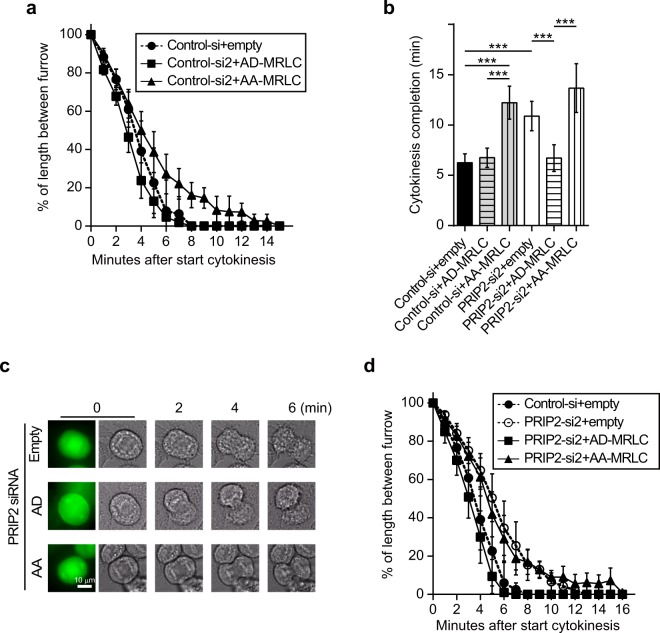
A phospho-mimic MRLC mutant restores delayed cytokinesis progression in *PRIP*-silenced HeLa cells. **(a**–**d)** Progression of furrow ingression was observed in HeLa cells transfected with the indicated siRNAs together with EGFP vector (empty), EGFP-AD-MRLC (AD-MRLC), or EGFP-AA-MRLC (AA-MRLC). The polygonal line graphs in **(a,d)** show calculation results; i.e., the measured width of the cleavage furrow divided by the initial width (0 min). The bar graphs in **(b)** represent the mean time to completion of furrowing in **(a,d)**. A set of representative time-lapse images is shown in **(c)**. Each experiment was performed at least three times **(a**–**d)**, and more than twenty images displaying similar features were obtained **(c)**. The data are presented as the means ± SD [n ≥ 20 for each group **(a,b,d)**]. ***p < 0.001 (Kruskal–Wallis test followed by Dunn’s multiple comparison test).

To verify that PRIP participates in pMRLC-mediated cytokinesis progression, we used two MRLC mutants, a phospho-mimic MRLC (AD-MRLC; amino acids Thr18 and Ser19 substituted with Ala and Asp, respectively) and a non-phosphorylatable form of MRLC (AA-MRLC; both Thr18 and Ser19 are substituted with Ala). EGFP-AA-MRLC localised to the cleavage furrow in HeLa cells (Supplementary Fig. S2), suggesting that localisation of MRLC to the cleavage furrow does not require Thr18/Ser19 phosphorylation. It was previously reported that transfection with exogenous AA-MRLC inhibited the phosphorylation of endogenous MRLC at the cleavage furrow^29^. We then analysed cytokinesis speed in normal HeLa cells and found that it was decreased in *AA-MRLC*-overexpressing cells, whereas it was similar in *AD-MRLC*-overexpressing and empty vector-transfected controls (Fig. 4a), suggesting that reducing the phosphorylation of MRLC inhibits cytokinesis speed.

We next examined whether the AA-MRLC and AD-MRLC mutants affect cytokinesis progression modulated by PRIP. The decrease in cytokinesis speed in *PRIP2*-depleted HeLa cells was not rescued by transfection with *AA-MRLC*. However, cytokinesis speed was restored to control siRNA-transfected cell levels upon transfection with *AD-MRLC* (Fig. 4b–d), indicating that PRIP modulates the upstream signalling of MRLC phosphorylation.

### PRIP is involved in RhoA-mediated cytokinesis

**Figure 5:**
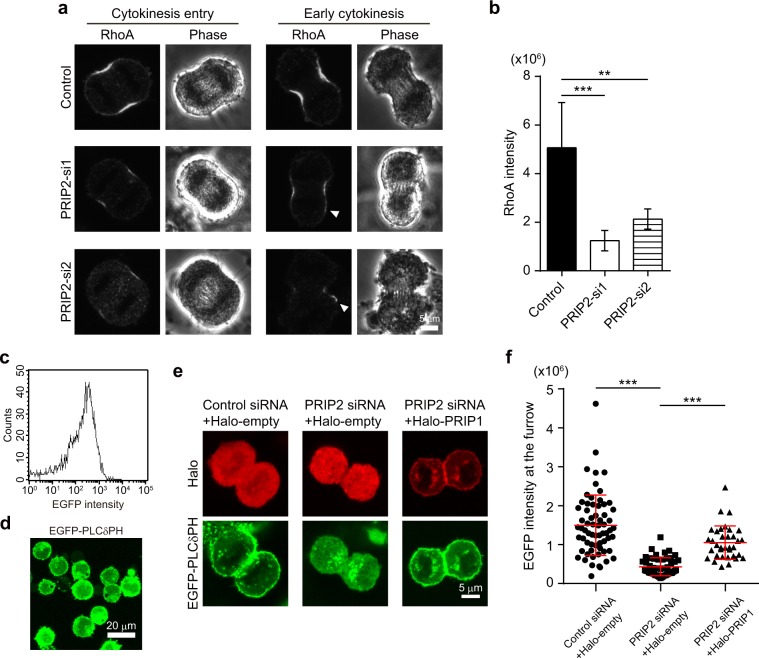
PRIP regulates the accumulation of RhoA and coordinates PI(4,5)P_2_ levels at the cleavage furrow. (**a**,**b**) Transfection with PRIP2-si1 or PRIP2-si2 inhibits RhoA accumulation at the cleavage furrow in HeLa cells. Representative images in a stage of cytokinesis (cytokinesis entry and early cytokinesis) are shown in **(a)**. RhoA signals were visualised using a specific antibody. Arrowheads in **(a)** indicate abnormal localisation of RhoA. Graph in **(b)** shows the fluorescence intensity (arbitrary units) of RhoA at the cleavage furrow in early cytokinesis. The data are presented as the mean ± SD (n = 45 for each group). **p < 0.01, ***p < 0.001 (Kruskal–Wallis test followed by Dunn’s multiple comparison test). **(c–f)** *PRIP2* silencing inhibits PI(4,5)P_2_ accumulation at the cleavage furrow in HEK293 cells. HEK293 cells stably expressing EGFP-tagged pleckstrin homology domain of PLCδ1 (EGFP-PLCδPH) were transfected with the indicated siRNAs **(e**,**f)** together with Halo-tagged vector (Halo-empty) or Halo-tagged *PRIP1* (Halo-PRIP1) **(e**,**f**). Analyses of EGFP intensity of the cells were performed by flow cytometry **(c)**. The cells were trypsinised and observed **(d)**. **(e**,**f)** Representative images of EGFP-PLCδPH and Halo-tagged proteins in a stage of cytokinesis are shown in **(e)**. Graph in **(f)** presents the mean intensity of EGFP in the cleavage furrow ± SD (n > 60 for each group). ***p < 0.001 (Kruskal–Wallis test followed by Dunn’s multiple comparison test).

Rho catalyses actin nucleation and polymerisation to form actin filaments and activates myosin cross-linking^30^. Downstream of Rho activation, RhoA induces mammalian homologue of *Drosophila* diaphanous (mDia)-mediated actin nucleation and polymerisation, as well as ROCK/pMRLC-dependent myosin II activation, consequently leading to the assembly and contraction of the actomyosin ring at the cleavage furrow^31,32^. *PRIP2*-knockdown HeLa cells exhibited a decrease in the thickness of F-actin as well as a reduced signal intensity of pMRLC in the cleavage furrow (Fig. 3a,b). Therefore, we wondered whether PRIP regulates the signalling of RhoA as an upstream regulator in cytokinesis progression. To this end, we investigated RhoA localisation at the cleavage furrow by immunocytochemistry with an anti-RhoA antibody. The RhoA signal was weaker at the cleavage furrows of both PRIP2*-*si1- and PRIP2-si2-transfected HeLa cells than that in control siRNA-transfected cells during the initiation and early steps of cytokinesis (Fig. 5a). In addition, we detected the aberrant localisation of RhoA in *PRIP2*-silenced cells (Fig. 5a, arrowheads). *PRIP2*-silenced cells showed a significant decrease in the fluorescence intensity of RhoA signal at the cleavage furrow (Fig. 5b). These results suggest that PRIP is necessary for the proper localisation of RhoA at the cleavage furrow.

### PRIP coordinates PI(4,5)P_2_ levels at the cleavage furrow

PI(4,5)P_2_ is necessary for correct furrow ingression during cytokinesis^5,8^. Rho1, the budding yeast RhoA homologue, binds to PI(4,5)P_2_ directly, which is required to complete cytokinesis^9^. In addition, PI(4,5)P_2_ regulates the accumulation of RhoA at the cleavage furrow in HeLa cells^3^. We previously reported a protective effect of PRIP on PI(4,5)P_2_ metabolism in migrating cells^27^. Therefore, we predicted that PI(4,5)P_2_ accumulation at the cleavage furrow would be regulated by PRIP. To directly monitor plasma membrane PI(4,5)P_2_ levels during cytokinesis, we successfully established a HEK293 cell line that stably expressed the EGFP-tagged PH domain of PLCδ1 (EGFP-PLCδPH), a probe for PI(4,5)P_2_. The isolated cell line had a single peak distribution of EGFP intensity, as determined by flow cytometry (Fig. 5c), and EGFP-PLCδPH signal was uniformly detected at the plasma membrane in trypsinised cells (Fig. 5d). Endogenous PRIP2 in the established HEK293 cells was successfully depleted to levels of about 40% and 20% by transfection with PRIP2-si1 and PRIP2-si2, respectively (Supplementary Fig. S3). *PRIP2* silencing dispersed the EGFP-PLCδPH signal from the cleavage furrow to the cytoplasm (Fig. 5e, left vs. middle panels, and Fig. 5f). However, upon co-transfection of Halo-*PRIP1* in *PRIP2*-silenced cells, Halo-PRIP1 signal was highly retained at the cleavage furrow, together with EGFP-PLCδPH signal (Fig. 5e, right panels, and Fig. 5f).

To further investigate the relationship between PRIP localisation at the cleavage furrow and PRIP binding to PI(4,5)P_2_, BODIPY FL-PI(4,5)P_2_ was exogenously added to HeLa cell culture before examination of PRIP localisation. BODIPY FL-PI(4,5)P_2_ signal was observed at the cleavage furrow and the plasma membrane, and in intracellular components (Supplementary Fig. S4). Consistently, DsRed2-PRIP1 and DsRed2-PRIP2 signals were co-localised with BODIPY FL-PI(4,5)P_2_ signal, including at the cleavage furrow. However, the signal of DsRed2-PRIP1(R134Q), in which the PH domain fails to bind PI(4,5)P_2_^33^, was observed in the cytoplasm and failed to accumulate at the cleavage furrow (Supplementary Fig. S4, panels on the far right). These results suggest that PRIP arrives to the cleavage furrow and is interdependently retained with PI(4,5)P_2_ in this location via the PH domain of PRIP.

### PRIP maintains PI(4,5)P_2_ at the cleavage furrow during cytokinesis

**Figure 6:**
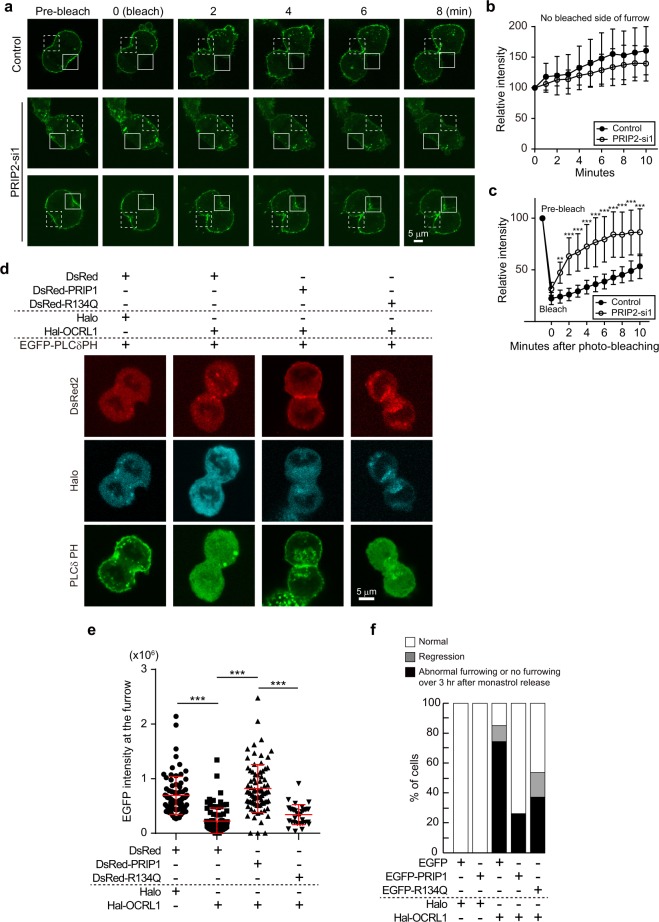
PRIP maintains PI(4,5)P_2_ at the cleavage furrow and appropriately manages PI(4,5)P_2_-dependent cytokinesis progression. **(a**–**c)** A time-lapse experiment using stably EGFP-PLCδPH-expressing HEK293 cells and fluorescence recovery after photobleaching (FRAP) analysis. The bleached area (or the pre-bleached area in 0–8 min panels) and non-bleached furrow region are outlined by a white square and a white dotted square on a set of representative images in **(a)**, respectively. Representative images of PRIP2-si1-treated cells are shown as two data sets, a delayed cytokinesis phenotype (middle panel set) and a slightly delayed cytokinesis phenotype (bottom panel set) compared with conventional cytokinesis (control, upper panel set). The graphs in **(b**,**c)** present the relative fluorescence intensity of EGFP-PLCδPH [normalised to the value at time 0 **(b)** or the pre-bleach value **(c)**] on the non-bleached side of the furrow **(b)** or bleached cleavage furrow **(c)** at each time point. The data are presented as the means ± SD (n > 20 for each group). **p < 0.01, ***p < 0.001 versus the control value at each time point (Kruskal–Wallis test followed by Dunn’s multiple comparison test). **(d**,**e)** HEK293 cells stably expressing EGFP-tagged PLCδPH were transfected with indicated constructs. Representative images at a stage of cytokinesis are shown in **(d)**. Graph in **(e)** shows the EGFP fluorescence intensity (arbitrary units) at the cleavage furrow. The data are presented as the mean ± SD (n > 45 for each group]. ***p < 0.001 (Kruskal–Wallis test followed by Dunn’s multiple comparison test). **(f)** MCF-7 cells stably expressing control vector (EGFP), EGFP-tagged PRIP, or EGFP-tagged PRIP1(R134Q) were co-transfected with Halo-tagged OCRL1 or Halo-tag vector. Quantitative analysis of cytokinetic abnormalities in OCRL1-transfected MCF-7 cells with indicated PRIP1 mutants were evaluated at 3 h after release from monastrol. Each experiment was repeated at least three times (n > 30 for each group).

Although PRIP is engaged in maintaining PI(4,5)P_2_ levels in the cleavage furrow (Fig. 5f), it remains unknown how PRIP regulates PI(4,5)P_2_ levels. We hypothesised that the mechanism by which PRIP regulates PI(4,5)P_2_ levels at the cleavage furrow is either via the upregulation of new PI(4,5)P_2_ synthesis or the downregulation of PI(4,5)P_2_ metabolism into other phospholipids. To address this issue, we examined the turnover of PI(4,5)P_2_ at the cleavage furrow using stably EGFP-PLCδPH-expressing HEK293 cells. The synthesis and metabolism rates of PI(4,5)P_2_ were assessed by measuring the GFP intensity of PLCδPH via fluorescence recovery after photobleaching (FRAP) analysis. We selected a cell starting cytokinesis and bleached the region of the cleavage furrow with a laser line. At the non-bleached side of the cleavage furrow, the GFP intensity did not differ between control siRNA- and PRIP2-si1-transfected cells (Fig. 6a,b), suggesting that the PI(4,5)P_2_ synthesis rate is similar between the two types of cells. By contrast, on the bleached side, the recovery of GFP intensity was slower in *PRIP*-expressing control HEK293 cells than in *PRIP2*-silenced cells (Fig. 6a,c), suggesting that PRIP maintains PI(4,5)P_2_ at the cleavage furrow and suppresses PI(4,5)P_2_ metabolism.

It is reported that silencing of *OCRL1* (an inositol polyphosphate 5-phosphatase) in HeLa cells results in the accumulation of PI(4,5)P_2_ at the intercellular bridge in late cytokinesis^34^. We ensured that Halo-*OCRL1* overexpression efficiently reduced PI(4,5)P_2_ levels at the intercellular bridge compared with that in control cells in late cytokinesis (Supplementary Fig. S5). Then, we used *OCRL1*-overexpressing cells to investigate whether PRIP modulates PI(4,5)P_2_ metabolism by OCRL1 at the cleavage furrow. We found that, during cleavage furrow formation, exogenously expressed Halo-OCRL1 was diffusely distributed in HEK293 cells, and PI(4,5)P_2_ levels (PLCδPH signal) were obviously reduced at the plasma membrane, including the cleavage furrow, compared with that in Halo vector-transfected control cells (Fig. 6d, compare first two panels). *Prip1* and Halo-*OCRL1*-co-transfected HEK293 cells exhibited the accumulation of PLCδPH signal with DsRed-PRIP1 signal (Fig. 6d, third panel). However, DsRed*-Prip1(R134Q)-*mutant and Halo-*OCRL1*-co-transfected HEK293 cells exhibited blurred signals of PLCδPH and DsRed-PRIP1(R134Q) (Fig. 6d, far right panel). The PI(4,5)P_2_ levels at the cleavage furrow were then assessed based on the intensity of the PLCδPH signal. *Prip1* overexpression in Halo-*OCRL1*-transfected HEK293 cells restored PI(4,5)P_2_ levels to those in non-Halo-*OCRL1*-transfected cells, but *Prip1(R134Q)* overexpression did not (Fig. 6e).

To investigate the effects of PRIP in OCRL1-expressed cells on cytokinesis progression, we performed live-cell imaging. We used MCF-7 cells stably expressing *Prip1*, *Prip1(R134Q)*, or EGFP vector^27^ in which Halo vector or Halo-*OCRL1* was transiently transfected. Approximately 80% of *OCRL1*-transfected EGFP vector-expressing MCF-7 cells showed abnormal cytokinesis (Fig. 6f). Importantly, these abnormal phenotypes were reduced in *OCRL1*-transfected *Prip1*-expressing cells and were partially restored in *OCRL1*-transfected *Prip1(R134Q)-*expressing cells. These results suggest that PRIP protects PI(4,5)P_2_ from OCRL1-mediated hydrolysis, which requires the binding of PRIP and PI(4,5)P_2_ via the PRIP PH domain.

### PRIP prevents PI(4,5)P_2_ recognition by PI(4,5)P_2_-metabolising enzymes OCRL1 and PLCδ1, but not by RhoA, *in vitro*

**Figure 7:**
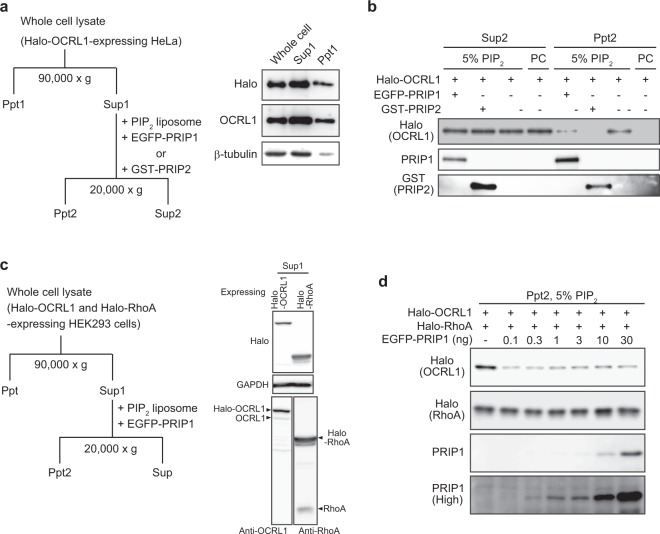
PRIP prevents PI(4,5)P_2_ recognition by OCRL1 but not by RhoA. **(a**–**d)** PI(4,5)P_2_ sedimentation assay was performed in a combination of EGFP-PRIP1, GST-PRIP2, Halo-OCRL1, and Halo-RhoA (**a**,**c**). Supernatant 1 (Sup1) contained Halo-OCRL1 **(a)** or Halo-OCRL1 and Halo-RhoA **(c)**, as confirmed by immunoblotting using indicated antibodies. The fractions of Sup1 in **(a** or **c)** precipitated with liposomes [PC, 100% PC; 5% PIP_2_, PI(4,5)P_2_:PC = 5:95 (molar ratio)] in the presence (+) or absence (−) of recombinant PRIP1 or PRIP2 **(b)** or with liposomes (5% PIP_2_) in the presence of indicated doses of recombinant PRIP1 **(d)** by centrifugation, respectively. Western blotting of the obtained pellet fraction (Ppt2, **b** or **d**) and supernatant (Sup2, **b**) was performed using indicated antibodies (left side of each panel). Similar data were obtained from three independent experiments, and a set of representative images is shown. The image obtained in a high sensitivity mode (high, **d**) is shown. Each of the original blots is shown in Supplementary Fig. S8 for **(d**–**f)** or Supplementary Fig. S9 for **(b)**.

We next examined whether PRIP affects the PI(4,5)P_2_ recognition of OCRL1 and PLCδ1, PI(4,5)P_2_-metabolising enzymes. An *in vitro* co-sedimentation assay was performed using liposomes composed of 100% phosphatidylcholine (PC) or 5% PI(4,5)P_2_ and 95% PC following the procedure described in Fig. 7a (for OCRL1) and Supplementary Fig. S6a (for PLCδ1). The supernatant (Sup1; Fig. 7a for OCRL1, Supplementary Fig. S6a for PLCδ1) was prepared from the whole-cell lysate of Halo-*OCRL1*-expressing or PLCδ1-expressing HeLa cells by centrifugation and used in the precipitation assays. Halo-OCRL1 precipitated with liposomes composed of 5% PI(4,5)P_2_ and 95% PC but did not precipitate with 100% PC liposomes (Fig. 7b, two lanes on the far right). Upon the addition of PRIP1 or PRIP2 to the assay tube, the association between Halo-OCRL1 and PI(4,5)P_2_ was inhibited (Fig. 7b). Similarly, PLCδ1 precipitated with liposomes composed of 5% PI(4,5)P_2_ and 95% PC. However, the association of PLCδ1 to PI(4,5)P_2_ was inhibited by the addition of PRIP1 (Supplementary Fig. S6b). These data suggest that PRIP, which lacks PLC activity, prevents the binding of PI(4,5)P_2_ to its metabolising enzymes such as OCRL1 and PLCδ1, thus negatively modulating PI(4,5)P_2_ metabolism.

RhoA also binds to PI(4,5)P_2_ and modulates PI(4,5)P_2_-mediated cytokinesis^9^. Therefore, to determine whether PRIP affects the association between RhoA and PI(4,5)P_2_, we performed the PI(4,5)P_2_-liposome sedimentation assay in the presence of OCRL1, RhoA, and PRIP1 (Fig. 7c). The association between OCRL1 and PI(4,5)P_2_ was inhibited by a low dose of PRIP. However, the association between RhoA and PI(4,5)P_2_ was not affected, even at a high dose of PRIP (Fig. 7d). These results suggest that PRIP affects the substrate recognition of OCRL1 but does not affect the association between RhoA and PI(4,5)P_2_.

## Discussion

Cytokinesis is a highly ordered process requiring an intricate interplay among cytoskeletal, chromosomal, and cell cycle regulatory pathways. Additional cellular processes, including phosphoinositide metabolism on the plasma membrane, are also important for cytokinesis. PI(4,5)P_2_, which is an integral signalling molecule despite being a minor component of cellular membranes, is generated by metabolic enzymes at the cleavage furrow and functions there during cytokinesis^35^. Therefore, adequate PI(4,5)P_2_ metabolic regulation is crucial for correct cytokinesis progression. The findings in this study demonstrated that the binding of PRIP to PI(4,5)P_2_ maintains normal PI(4,5)P_2_ levels at the cleavage furrow, which leads to the proper completion of cytokinesis. We thus propose that PRIP is a new candidate for protecting PI(4,5)P_2_ from metabolism by associated enzymes during cytokinesis. Elucidation of this novel PRIP-mediated cytokinesis regulation pathway may aid our understanding of fundamental cellular mechanisms.

PI(4,5)P_2_ interacts with Rho, regulates Rho GTPase activity at the cleavage furrow, and facilitates the recruitment of contractile ring constituents to the cleavage furrow as a scaffold lipid^3,9^. We previously reported that PRIP negatively regulates PI(4,5)P_2_ metabolism in migrating cells^27^. In this study, a deficiency of *PRIP2* in HeLa cells was found to cause cytokinesis defects and delay by suppressing the accumulation of PI(4,5)P_2_ and RhoA at the cleavage furrow. The demonstrated PRIP role here is that PRIP maintains PI(4,5)P_2_ levels by protecting PI(4,5)P_2_ from its metabolising enzymes at the cleavage furrow and regulates the RhoA/pMRLC-dependent progression of cytokinesis. Rho also stimulates PIP5K activity^36^ and ROCK-mediated phosphatase and tensin homologue (PTEN) signalling^37^, which results in the upregulation of PI(4,5)P_2_ synthesis. Thus, PI(4,5)P_2_, under the guardianship of PRIP, may accelerate the accumulation of RhoA at the cleavage furrow, followed by further PI(4,5)P_2_ production by PI5K and/or PTEN. This positive feedback loop contributes to the initiation of the cleavage furrow and the promotion of cleavage furrow ingression to complete cytokinesis.

Anillin, a highly concentrated protein in the cleavage furrow, is a scaffolding protein that links RhoA, F-actin, and myosin II and regulates the contractile ring^31^. RhoA activates ROCK and citron kinase, which phosphorylate the MRLC of non-muscle myosin II^31^. RhoA also binds and activates formins that elicit actin nucleation and polymerisation. Thus, RhoA coordinates the RhoA/ROCK/pMRLC and RhoA/formin signalling pathways and facilitates actomyosin ring assembly and contraction. In this study, *PRIP2*-silenced cells exhibited reduced pMRLC levels and the accumulation of F-actin and RhoA at the cleavage furrow. Therefore, these results indicate that PRIP is the most upstream molecule in the RhoA-mediated signalling pathways, profoundly modulating F-actin formation and MRLC phosphorylation. Although *PRIP2*-silenced cells displayed reduced accumulation of F-actin at the cleavage furrow, the transfection of a phospho-mimic MRLC mutant (AD-MRLC) alone efficiently rescued the cytokinesis delay in *PRIP2*-silenced cells. It was previously reported that actomyosin contractile ring assembly is regulated by actin-related protein 2 and 3 (ARP2/3), which enhance actin nucleation^38,39^. Myosin II heavy chain accumulates at the cleavage furrow and promotes the elongation of actin bundles^29,40^. These are independently modulated by the RhoA pathway during cytokinesis. Therefore, regulation of F-actin assembly at basal levels during cytokinesis is most likely modulated by RhoA-independent signalling.

PI(4,5)P_2_ is a substrate of PLC, OCRL1, and PI3K. These enzymes are involved in PI(4,5)P_2_ turnover. Several PLC isoforms are localised to the cleavage furrow, and PLC activity is required for cytokinesis^41,42^. The overexpression of synaptojanin failed to lead to PI(4,5)P_2_ and RhoA accumulation in the cleavage furrow^3^. In addition, a PI(4,5)P_2_ phosphatase, OCRL, which is mutated in patients with oculocerebrorenal syndrome of Lowe, participates in cell abscission during cytokinesis^34^. Inhibition of the PI(4,5)P_2_ kinase PI3K by a PI3K inhibitor such as LY294002 or wortmannin induces a delay in cytokinesis when the inhibitor is added at during furrow cleavage formation^43^. These reports indicate that the dynamic regulation of PI(4,5)P_2_ metabolism and PI(4,5)P_2_ maintenance at the cleavage furrow are crucial for correct cytokinesis progression. Here, we showed that the overexpression of OCRL1 resulted in reduced PI(4,5)P_2_ accumulation at the cleavage furrow and abnormal and delayed cytokinesis. This phenotype was rescued by the additional expression of PRIP1 but not of a PI(4,5)P_2_-unbound PRIP mutant. Importantly, FRAP analysis confirmed the inhibitory effect of PRIP on PI(4,5)P_2_ metabolism at the cleavage furrow. Collectively, our data demonstrate that PRIP protects PI(4,5)P_2_ via the PRIP PH domain from metabolism by its enzymes and maintains sufficient levels of PI(4,5)P_2_ for cytokinesis.

RhoA has a carboxy-terminal polybasic tail, which can, through electrostatic interactions, directly bind to several phosphatidylinositols, including PI(4,5)P_2_, *in vitro*. This binding activity requires the recruitment or stabilisation of RhoA at the cleavage furrow^9,10,44^. In our experiments, PRIP competitively inhibited substrate recognition by OCRL1 or PLCδ1 but did not affect the binding of RhoA to PI(4,5)P_2_. These data indicate that the binding mode of RhoA to PI(4,5)P_2_ differs from those of PRIP, OCRL1, and PLCδ1. These results support the positive role of PRIP in regulating the PI(4,5)P_2_/RhoA signalling pathway.

PRIP contains an X-Y domain, which is similar to the PLC catalytic core subunit in PLCδ1. Although PRIP1 and PRIP2 do not have activity, the X-Y domain of PRIP can associate with PI(4,5)P_2_^15,27^. PRIP1(R134Q), a PI(4,5)P_2_-unbound mutant with a mutation in the PH domain^33^, slightly accumulated at the cleavage furrow and slightly rescued cytokinesis abnormalities observed in *OCRL1*-overexpressing cells. The binding of the X-Y domain to PI(4,5)P_2_ in the PRIP(R134Q) mutant may inhibit the association between PI(4,5)P_2_ and OCRL1. Consequently, the PRIP(R134Q) mutant partially restored cytokinesis.

This is the first study to elucidate the existence of a rheostat for PI(4,5)P_2_ metabolism in the cleavage furrow during cytokinesis. Our findings conclusively demonstrate that, even if PI(4,5)P_2_ is exposed to a number of its metabolic enzymes, PRIP can maintain the high levels of PI(4,5)P_2_ required at the cleavage furrow to promote correct cytokinesis. The continuous phosphoinositide cycling required at the cleavage furrow is regulated by a positive feedback mechanism involving PI(4,5)P_2_ accumulation via PRIP-mediated RhoA signalling, which is necessary for normal cytokinesis progression.

## Methods

### Plasmids and siRNAs

EGFP-tagged *PLCδPH*, *Prip1*, *Prip1(R134Q)*, and *PRIP2*; DsRed2-tagged *Prip1* and *Prip1(R134Q);* GST-tagged *PRIP2* and *PLCδ1*; and Halo-*PRIP1* were described previously^17,23,45^. EGFP-tagged *AD-MRLC* and *AA-MRLC* were described previously^46^. Halo-tagged *OCRL1* (FHC01740) and *RHOA* (FHC01522) were purchased from Promega (Madison, WI, USA). Human *PRIP2*-siRNAs (si1, 2023-007 and 2023-008; si2, 2023-009 and 2023-010) were purchased from Sigma Genosys (Woodlands, TX, USA). A negative control siRNA (S10C-0600) was purchased from Cosmo Bio (Tokyo, Japan).

### Antibodies

Anti-phospho-MRLC (Ser19; #3671), anti-GAPDH (#2118), anti-OCRL1 (#8797), HRP-conjugated anti-rabbit IgG (#7074), and HRP-conjugated anti-mouse IgG (#7076) antibodies were purchased from Cell Signaling Technology (Beverly, MA, USA). Anti-PRIP1 and anti-PRIP2 polyclonal antibodies were developed previously^47–49^. Anti-β-actin antibody (IMG-5142A) was obtained from Imgenex (San Diego, CA, USA). Anti-β-tubulin (D66) and anti-total MRLC (MY-21) antibodies were purchased from Sigma-Aldrich (St. Louis, MO, USA). Anti-GST antibody (M209-3) was obtained from Medical & Biological Laboratories (Nagoya, Japan). Anti-RhoA (sc-418) and anti-PLCδ1 (D-7) antibodies were purchased from Santa Cruz Biotechnology (Dallas, TX, USA). Anti-HaloTag antibody (G928A) was purchased from Promega. Alexa Fluor 488-conjugated antibodies [anti-rabbit IgG (A11008), anti-mouse IgG (A11001), and anti-mouse IgM (A21042)], Alexa Fluor 594-conjugated anti-mouse IgG (A11005), and Alexa Fluor 405-conjugated antibodies [anti-rabbit IgG (A31556) and anti-mouse IgG (A31553)] were obtained from Invitrogen (Carlsbad, CA, USA). Alexa 594-conjugated anti-mouse IgM (21044) antibody was purchased from Thermo Fisher Scientific (Rockford, IL, USA).

### Cell culture and transfection

MCF-7 cells stably expressing EGFP-PRIP1 or EGFP-PRIP1(R134Q) were developed previously^27^. HEK293 cells were transfected with an expression vector encoding EGFP-PLCδPH or a control EGFP vector and cultured in the presence of 1 mg/ml G418 (Nakalai Tesque, Kyoto, Japan) for 14 days. Stably EGFP-expressing colonies were selected. Cells were cultured under conventional growth conditions and transfected with plasmids or siRNA using the Lonza 4D-Nucleofector X Unit (Lonza, Basel, Switzerland) or Lipofectamine 3000 (Invitrogen).

### Reverse transcription-PCR analysis

A 5-μg sample of total RNA from HeLa cells, isolated using an RNeasy minikit (Qiagen, Hilden, Germany), was used for reverse transcription reactions using a cDNA synthesis kit (Takara, Shiga, Japan). *PRIP1*, *PRIP2*, and *GAPDH* were amplified using specific primer pairs whose sequences were previously described^27^. The PCR products were distinguished by agarose gel electrophoresis.

### Analyses of living cells and FRAP

Cells were seeded on μ-dishes (Ibidi, Martinsried, Germany) and cultured until attached. Transfection was performed as described above. Cells were synchronised with 50 μM monastrol (Cayman Chemical, Ann Arbor, MI, USA) for 12 h, and then monastrol was washed out. For detection of HaloTag, cells were incubated with 1 μM of HaloTag TMR ligand or HaloTag Coumarin ligand (Promega) for 1 h before washout of the ligand. The cells were recorded every minute for 3 h by live-cell imaging on a BZ-9000 microscope (Keyence, Osaka, Japan). During FRAP analysis, EGFP images were scanned before and after bleaching at low laser power on a confocal laser scanning microscope (Fluoview FV10i; Olympus, Tokyo, Japan). To destroy EGFP fluorescence, maximal laser power was applied to a furrowing region for 30 s. Images were taken before and after bleaching at intervals of 1 min until completion of cytokinesis. Cytokinesis analysis and measurement of fluorescent intensity were performed using ImageJ 1.45 s (National Institutes of Health, Bethesda, MD, USA) following previously described methods^29^.

### Immunofluorescence and western blotting

Immunofluorescence and western blotting were carried out following previously described methods^50,51^. F-actin was visualised by ActinRed^TM^ 555 ReadyProbes (R37112) (Thermo Fisher Scientific) or Alexa Fluor 350-labelled phalloidin (Invitrogen). RhoA staining was carried out following the previously described method^52^. Fluoview FV10i was used for observations. The acquired images were analysed by ImageJ 1.45 s.

### Liposome sedimentation assay

A binding assay of OCRL1 and RhoA to liposomes containing PI(4,5)P_2_ was carried out using the method described previously^27^, with modifications, using PI(4,5)P_2_ (Cayman Chemical) and/or phosphatidylcholine (PC; Sigma-Aldrich). Cell lysates were obtained from cells transiently expressing Halo-OCRL1 and/or Halo-RhoA. Recombinant EGFP-PRIP1 and GST-PRIP2 were purified from MCF-7 cells stably expressing EGFP-*Prip1* and HEK293 cells transiently expressed GST-PRIP2. The detailed method is described in Fig. 7a,c.

### Statistical analysis

GraphPad Prism (GraphPad Software, La Jolla, CA, USA) was used for statistical analyses. The non-parametric Kruskal–Wallis test followed by Dunn’s multiple comparison test was used. A *p-*value less than 0.05 was considered statistically significant.

## Supplementary information

Supplementary Information

## Data Availability

All data generated or analysed during this study are included in this published article and its Supplementary Information file. Other relevant information is available from the authors on reasonable request.
