# Supplementary material for: Phospholipase C-related catalytically inactive protein regulates cytokinesis by protecting phosphatidylinositol 4,5-bisphosphate from metabolism in the cleavage furrow
Source: Sci Rep. 2019 Sep 4;9:12729. doi: 10.1038/s41598-019-49156-3 (PMC6726632; doi:10.1038/s41598-019-49156-3)
Supplement: Supplementary file 1 — Supplementary Information [file 41598_2019_49156_MOESM1_ESM.pdf]

Supplemental Information

Phospholipase C-related catalytically inactive protein regulates cytokinesis by protecting phosphatidylinositol 4,5-bisphosphate in the cleavage furrow

Satoshi Asano, Yasuka Ikura, Mitsuki Nishimoto, Yosuke Yamawaki, Kozue Hamao, Keiju Kamijo, Masato Hirata and Takashi Kanematsu

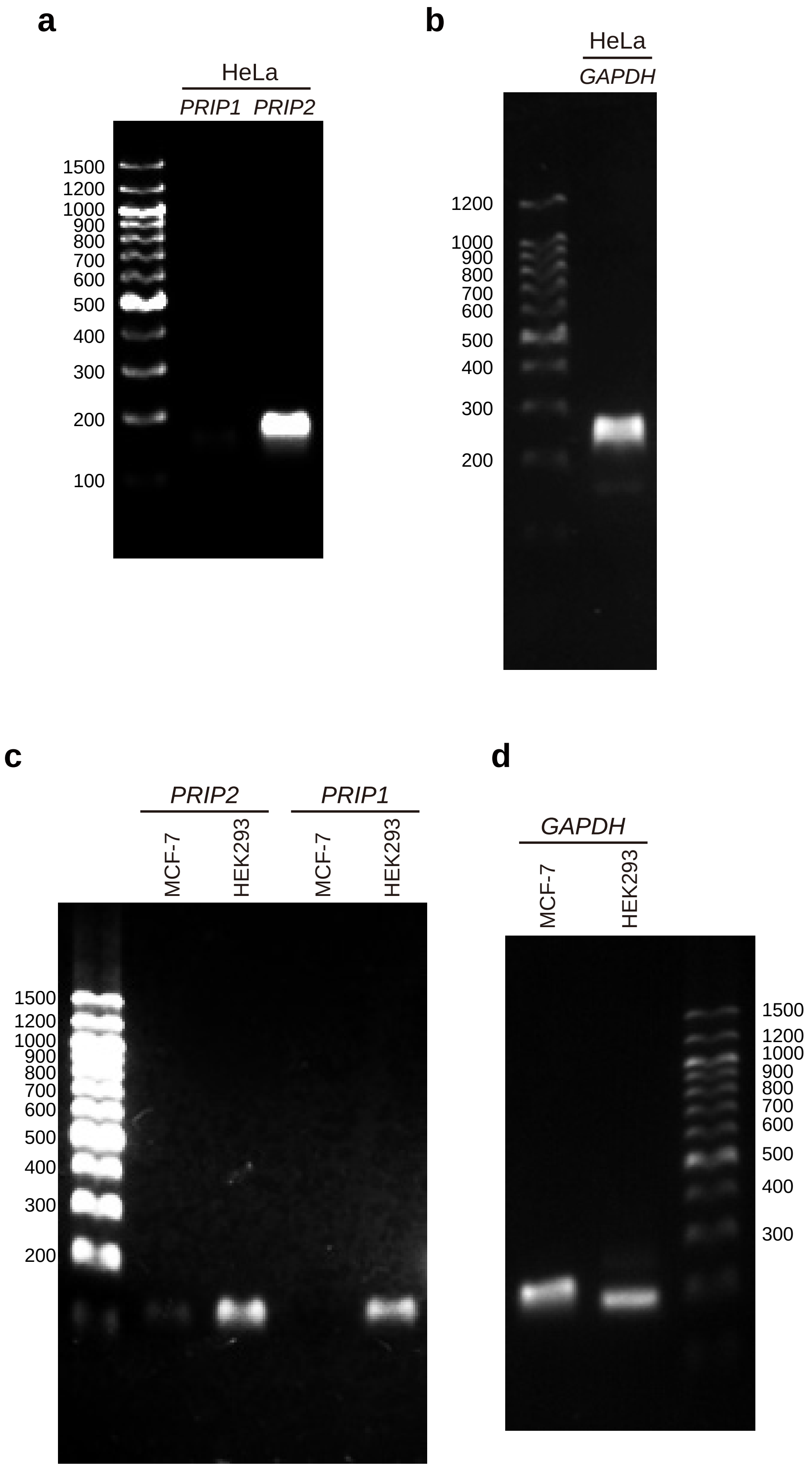

Supplementary Figure S1. (a–d) *PRIP1* and *PRIP2* gene expression determined by reverse transcription-PCR analysis (conducting for 40 cycle in HeLa **(a)**, MCF-7 **(c)** and HEK293 **(c)** cells. The nucleotide size of markers (base pair) is shown in numbers. Glyceraldehyde 3-phosphate dehydrogenase (GAPDH) is used as an internal control.

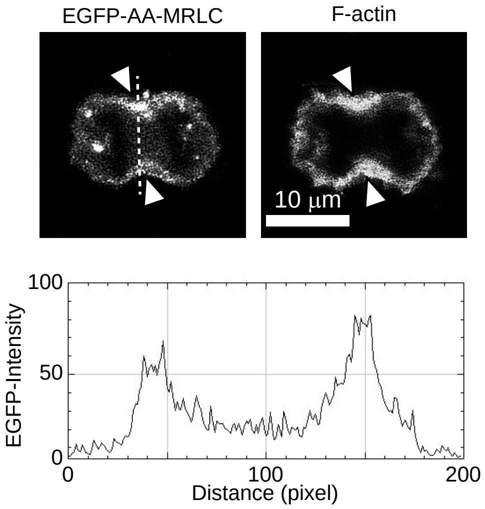

Supplementary Figure S2. Introduction of a non-phosphorylatable form of MRLC does not inhibit the localisation of F-actin formation at the cleavage furrow in HeLa cells. Cells were transiently transfected with an EGFP-tagged non-phosphorylatable form of *MRLC*, having T18A, S19A mutations (AA-MRLC). F-actin was visualised by Alexa Fluor 350-labelled phalloidin (Invitrogen). Arrowheads indicate the cleavage furrow. The fluorescence intensity of EGFP across the cell was obtained by a line scan at the dotted white line and is shown in the lower panel with arbitrary units. The experiment was repeated three times, and similar results were obtained.

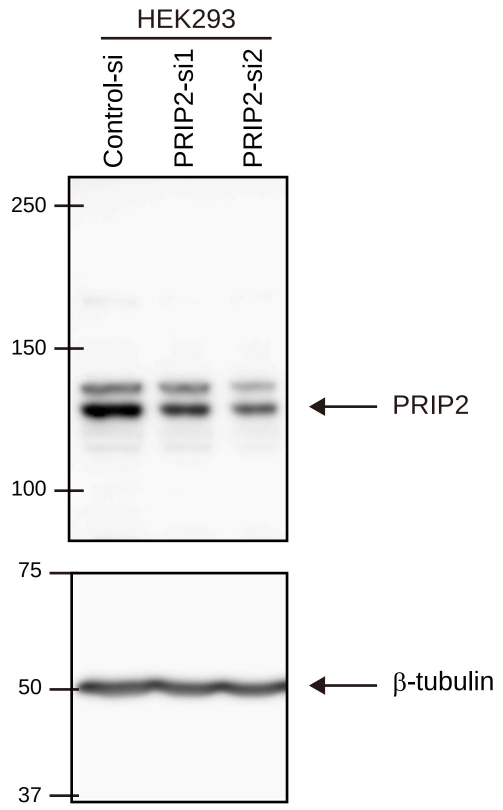

Supplementary Figure S3. (a,b) Success of PRIP2-silencing in stably EGFP-PLCδPH-expressing HEK293 cells analysed by western blotting. β-tubulin was used as a loading control. Control siRNA (Control-si) and PRIP2 siRNAs (PRIP2-si1 and PRIP2-si2) were used. Molecular-weight size (kDa) for protein markers is shown in numbers.

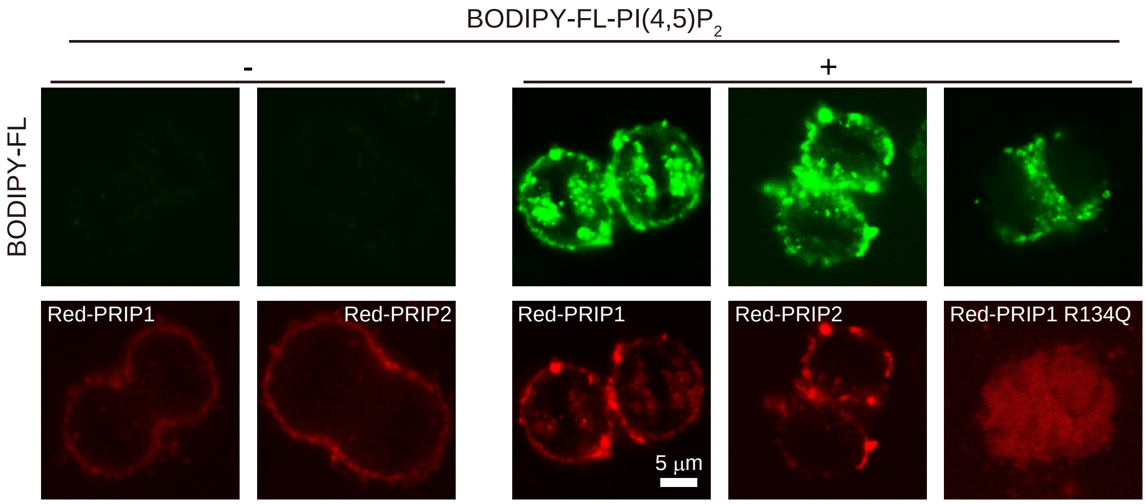

Supplementary Figure S4. $PI(4,5)P_2$ is involved in PRIP localisation at the cleavage furrow during cytokinesis. HeLa cells were transfected with PRIPs or PRIP1 R134Q. Exogenously added BODIPY FL-$PI(4,5)P_2$ (10 μM) accumulates transfected PRIP but not PRIP1 R134Q to the furrowing compartment in HeLa cells.

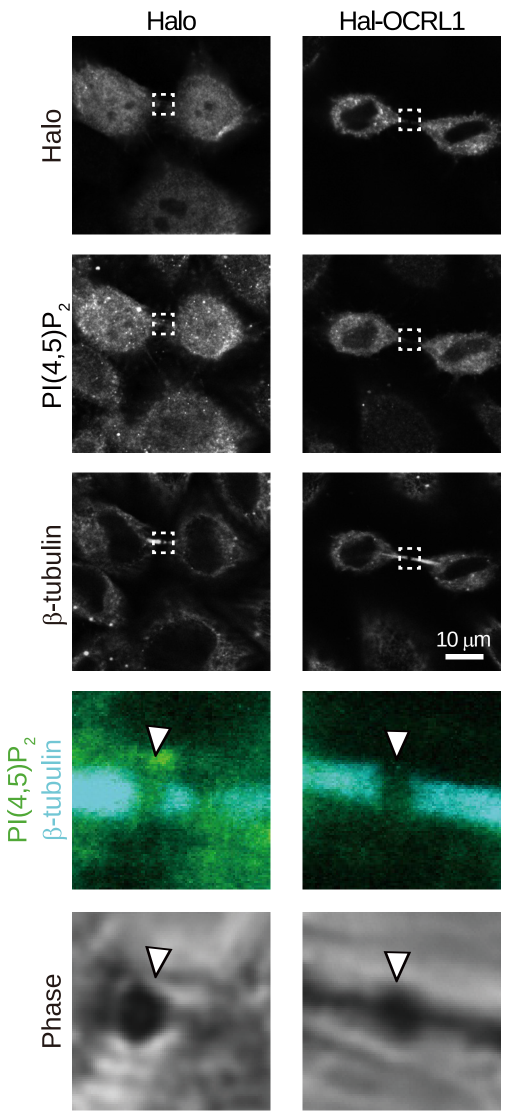

Supplementary Figure S5. Abnormal local accumulation of PI(4,5)P_2 at intercellular bridges in *OCRL1*-transfected HeLa cells. The localisation of PI(4,5)P_2 to intercellular bridges in HeLa cells transfected with Halo-tagged *OCRL1*. A set of fluorescence images was obtained at 2 h 30 min after release from monastrol. PI(4,5)P_2 was detected by anti-PI(4,5)P_2 antibody. The magnified view of the dotted boxed region is shown in the lower panels. Arrowheads indicate a midbody. Each experiment was repeated three times, and similar results were obtained.

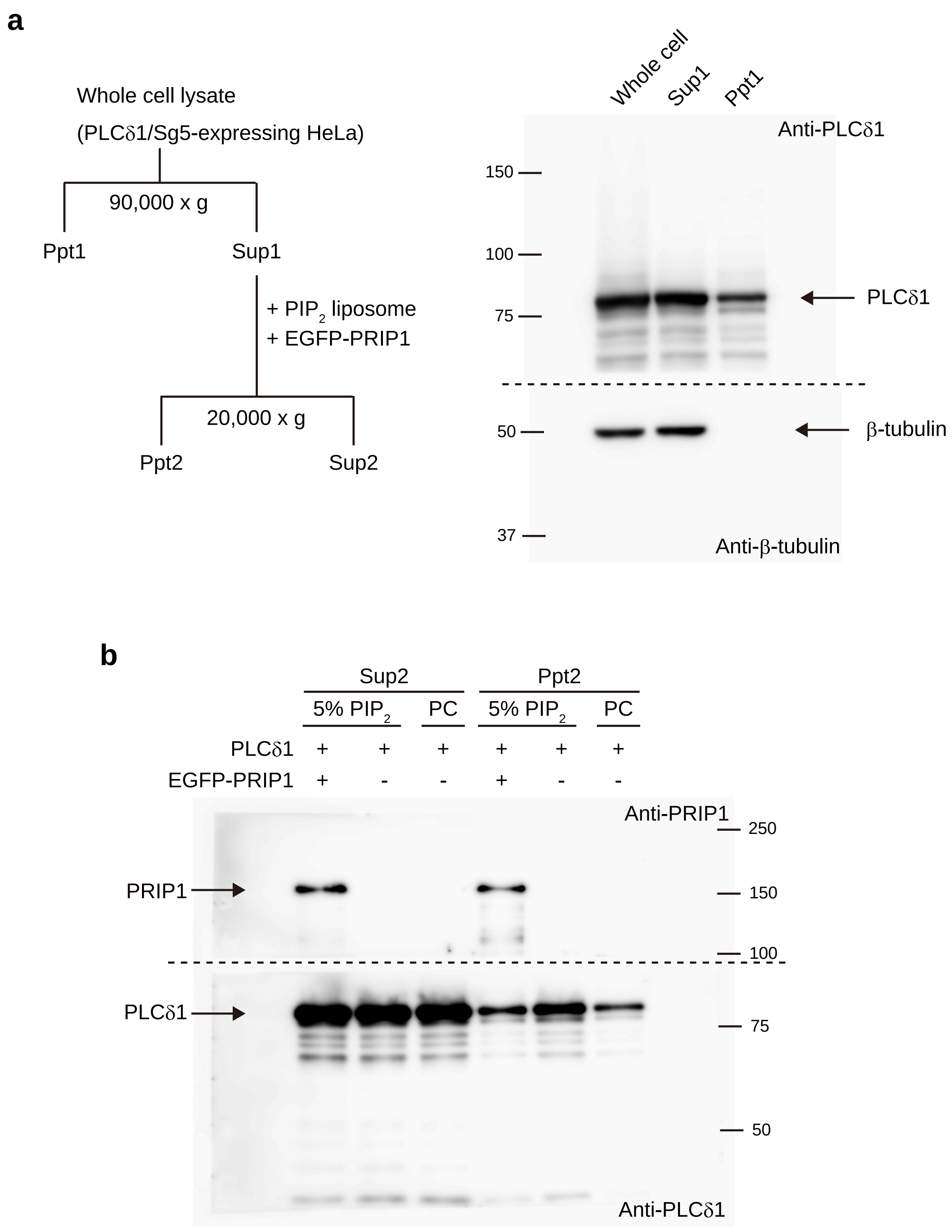

Supplementary Figure S6. PRIP suppresses binding between PLCδ1 and PI(4,5)P_2. **(a,b)** A PI(4,5)P_2 sedimentation assay was performed using PLCδ1-expressing HeLa cell lysate (**a**, Sup1 in the right panel) in the presence (+) or absence (−) of recombinant PRIP1. The method is schematically presented on the left in **(a)**. Liposomes are composed of PI(4,5)P_2: PC = 5:95 (molar ratio) (PIP_2) or 100% PC (PC). The obtained lipid fractions (Ppt2) and supernatants (Sup2) were evaluated by western blotting using an anti-PLCδ1 antibody and anti-PRIP1 antibody. The upper and lower membranes, which were separated at the dotted line, were used for the detection of PLCδ1 and β-tubulin in **(a)**, and PIRP1 and PLCδ1 in **(b)**, respectively. Molecular-weight size (kDa) for protein markers is shown in numbers. The experiment was repeated two times, and similar results were obtained.

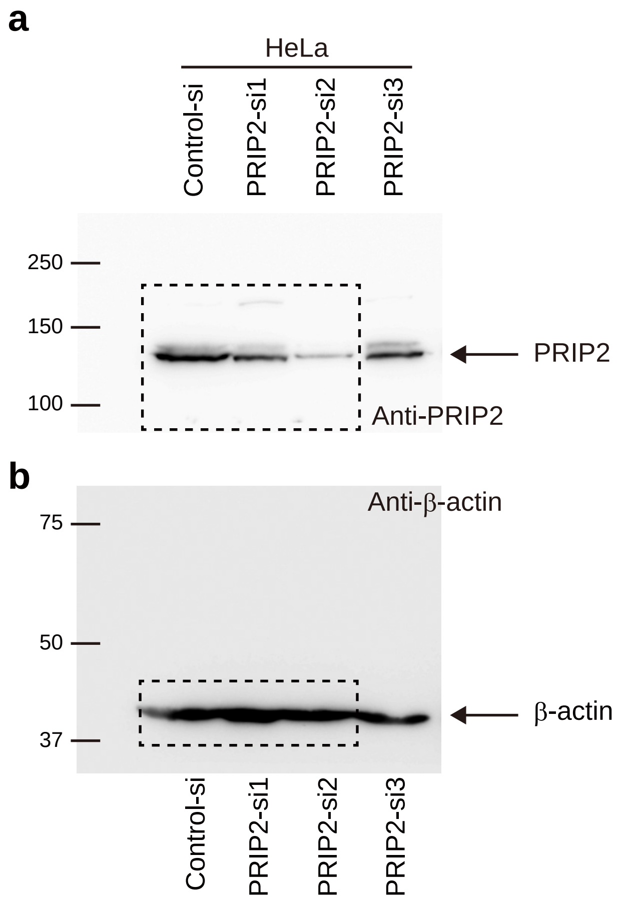

Supplementary Figure S7. (a,b) Full images of the crop blots used in Fig. 2a. Western blot analysis using the indicated antibodies was performed using a part of blotted membrane. The full image and cropped area represented in a dotted square are shown. Molecular-weight size (kDa) for protein markers is shown in numbers.

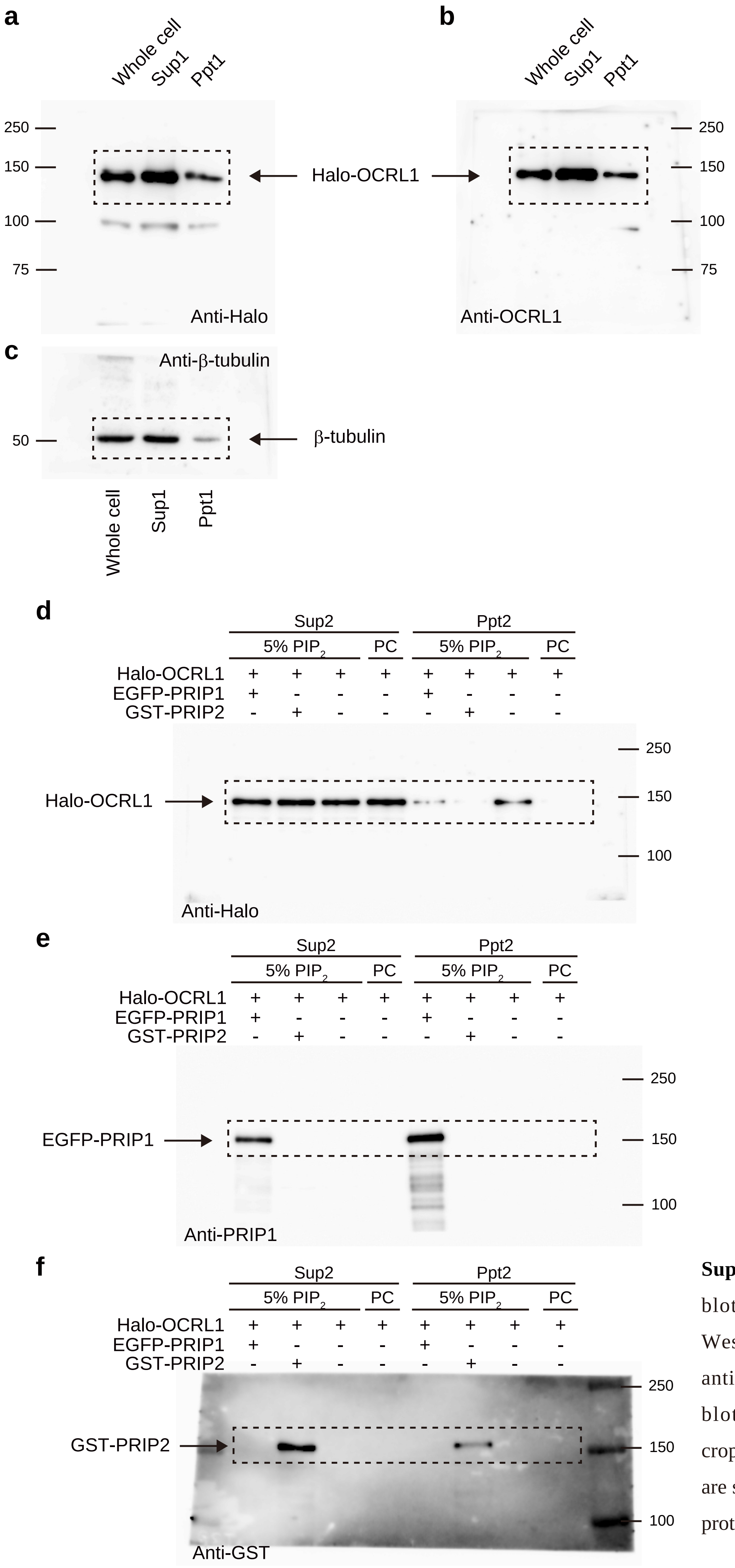

Supplementary Figure S8. (a–f) Original blots used in Fig. 6c **(a-c)** and 6d **(d-f)**. Western blot analysis using the indicated antibodies was performed using a part of blotted membrane. The full image and cropped area represented in a dotted square are shown. Molecular-weight size (kDa) for protein markers is shown in numbers.

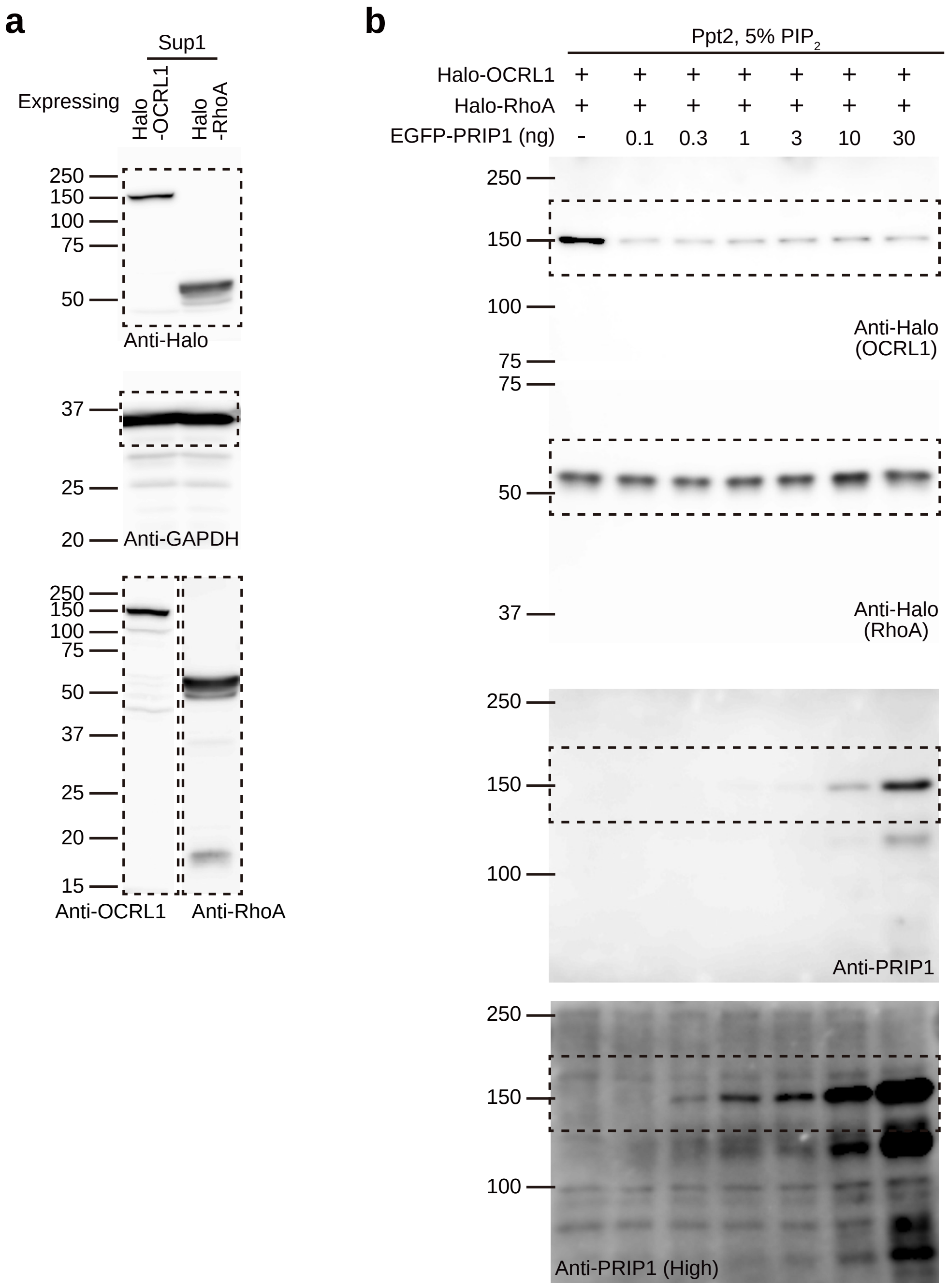

Supplementary Figure S9. (a,b) Original blots used in Fig. 6e **(a)** and 6f **(b)**. Western blot analysis using the indicated antibodies was performed using a part of blotted membrane. The full image and cropped area represented in a dotted square are shown. The bottom panel in **(b)** was obtained using a high sensitivity mode (High). Molecular-weight size (kDa) for protein markers is shown in numbers.
